# Bioactive polypeptide improves neuroinflammation by regulating microglia polarization

**DOI:** 10.1111/jcmm.17059

**Published:** 2021-12-30

**Authors:** Liping Zhai, Heping Shen, Yongjia Sheng, weiqun Guo, Qiaobing Guan, Yu Zhu

**Affiliations:** ^1^ The Second Affiliated Hospital of Jiaxing University Jiaxing China

## 1 BACKGROUND

Toll‐like receptor (TLR) was first discovered during a research of embryonic development in *Drosophila*.^1^ As a class of natural immune receptors, TLRs can identify pathogen‐associated molecular patterns (PAMP)^2^ to initiate relevant signalling pathways, which play a crucial role in immune responses and inflammation. Aβ is one major pathogenic protein of Alzheimer's disease (AD).^3^ Its cerebral accumulation can stimulate the intracellular signalling cascade via TLRs on the microglial surface, thereby activating microglia. The microglia can clear Aβ through phagocytosis and release pro‐inflammatory cytokines and cytotoxic substances.^4^ In AD, microglia polarize to pro‐inflammatory M1 phenotype upon stimulation. Such change is also mediated by TLR4 signal,^5^ with the precise mechanism being linked to NF‐κB downstream of TLR4. Thus, TLR4 has always been considered a therapeutic target for the AD‐related neuroinflammation.^6^, ^7^

Polypeptides derived from plants are diverse in type and varied in function, which, such as natural small‐molecular compounds, possess many pharmacological activities. It has been reported that RBAP, a rice‐derived bioactive polypeptide, can target TLR4 and resist the endothelial inflammation induced by hydrogen peroxide, which thus inhibits the TLR4 and downstream signals.^8^ In spite of these, the polypeptide has seldom been applied. Hence, this study further explores the role of RBAP in regulating the microglia polarization in AD by targeting TLR4.

## 2 MATERIAL AND METHODS

### 2.1 Effects of RBAP on M1 polarization of BV2 cells

The mouse microglia BV2 cells (Procell Life Science & Technology, Wuhan, China) were thawed and cultured in a 10% FBS‐containing complete medium at 37 °C with 5% CO_2_. After logarithmic growth with cell viability >80% upon trypan blue staining, the cells were passaged. We divided the cells into the control, the RBAP^8^ (Lys‐His‐Asn‐Arg‐Gly‐Asp‐Glu‐Phe, synthesized by our laboratory), the LPS/IFN‐γ(L/I) and the L/I+RBAP groups. The control cells were cultured routinely. The RBAP group was treated with 0.1 mM RBAP. In the L/I group, 100 ng/ml LPS (Sigma, USA) and 30 ng/ml recombinant IFN‐γ (KALANG, USA) were used to induce the M1 polarization of BV2 cells.^9^, ^10^ In the L/I+RBAP group, the M1 polarization was induced by LPS/IFN‐γ following 5‐h pretreatment with 0.1 mM RBAP, in order to observe the effects of RBAP on the BV2 and M1 BV2 cells.

Enzyme‐linked immunosorbent assay (ELISA): After BV2 cells were polarized by LPS/IFN‐γ for 48 h, the culture medium was obtained by centrifugation to measure the levels of TNF‐α, IL‐1β and IL‐6 (marker cytokines for M1 BV2 cells), as well as the levels of IL‐10 and TGF‐β1 (marker cytokines for M2 BV2 cells). The standard curve method was employed to perform calculations, and the results were expressed in ng/ml.

### 2.2 Effects of RBAP on neuroinflammation and microglia polarization in APP/PS1 AD mice

After completion of the behavioural tests, the mice were sacrificed by carbon dioxide asphyxiation, and their cerebral tissues were perfused with 4% paraformaldehyde for fixation.

1. IFC: For determination of cortical expressions of CD86 and CD206 in mice, the cerebral tissues were dehydrated with 15% and 30% sucrose solutions, embedded in OCT, sliced continuously into 8‐μm sections using a freezing microtome and kept at −20°C for subsequent use. After washing with PBS and blocking with 5% serum for 30 min, the sections were incubated with IBA‐1 monoclonal antibody at 4°C overnight and then washed 3 times with PBS. An extra 1‐h incubation with fluorescent antibody was carried out in the dark followed by 3 times washing with PBS. Finally, the sections were sealed with anti‐fluorescent quencher and observed microscopically.
2. ELISA: For measurement of cerebral tissue levels of M1 cell markers TNF‐α, IL‐1β and IL‐6, as well as M2 cell markers IL‐10 and TGF‐β1, the orbital peripheral blood was collected from the mice, and then, the mice were sacrificed by carbon dioxide asphyxiation. After homogenization of the hippocampal, cortical and striatal tissues, they were lysed on ice with RIPA lysate for 30 min and then quantified by BCA assay. The tissue protein supernatant and peripheral blood serum were collected and assessed by ELISA kit, and the results were expressed in ng/ml.

### 2.3 Statistical methods

All measurement data were presented as (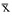
x ±s), and the SPSS 17.0 was used for statistical analysis and processing. After the homogeneity test of variances, comparisons between two sets of data were made by two independent samples t‐test, while comparisons among three or more sets of data were accomplished by one‐way ANOVA. Subsequent pairwise comparisons between the groups were all performed by LSD method. All the aforementioned tests were two‐sided, and *p* values of <0.05 were considered significantly different.

## 3 RESULTS

### 3.1 RBAP inhibits M1 polarization of BV2 cells

**FIGURE 1:**
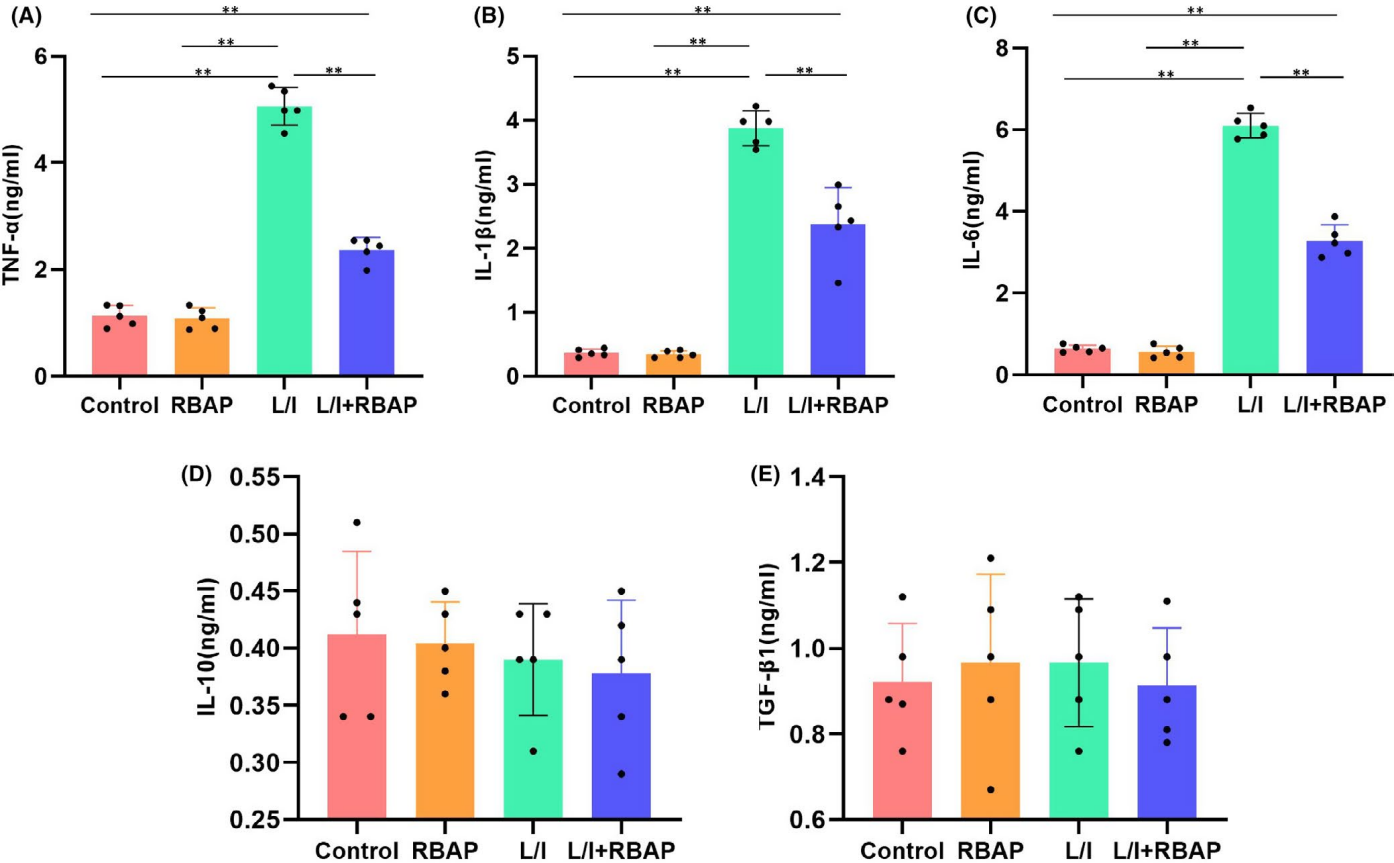
Effects of RBAP on the BV2 cell polarization markers. Regarding inflammatory cytokine detection, L/I could promote the inflammatory cytokine up‐regulation, exhibiting significantly higher cytokine levels than the Control group. Contrastively, RBAP could inhibit the inflammatory cytokine expressions. Inter‐group comparisons, **p < 0.05. According to M2 marker detection, RBAP showed insignificant effect on the expression of TGF‐β1 or IL‐10

During cytokine detection, L/I was found to promote the up‐regulation of inflammatory cytokine expressions, exhibiting significantly higher cytokine levels than the control group, while RBAP could inhibit the inflammatory cytokine expressions (Figure 1A‐C). Nevertheless, RBAP produced unobvious effect on the expression of TGF‐β1 or IL‐10, the M2 cell markers (Figure 1D‐E).

### 3.2 Effects of RBAP on behavioural patterns and microglia polarization in APP/PS1 mice

**FIGURE 2:**
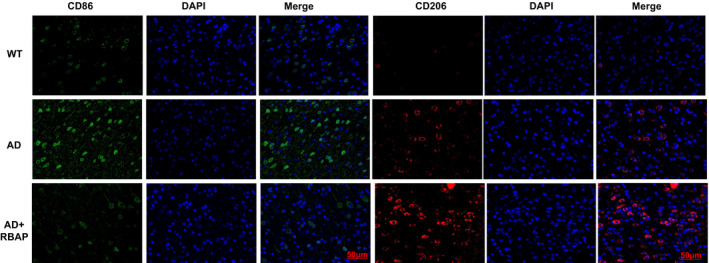
Effects of RBAP on microglia polarization in AD mice. IFC analysis revealed that in the WT mice, both the CD86 and CD206 levels were low, and the microglia were in a resting state. In the AD mice, CD86 expression was significantly up‐regulated, while the CD206 level was low. RBAP treatment could prominently inhibit the CD86 expression and up‐regulate the CD206 level

We tested the CD86 and CD206 expressions and found that both CD86 and CD206 levels were low in WT mice, indicating that the microglia in these mice were in a resting state. Contrastively, the CD86 level was significantly up‐regulated in AD mice, while the CD206 level was low. This indicates that the M1 type microglia were dominant in these mice, which also constitutes one major cause of neuroinflammation. After treatment with RBAP, the CD86 expression could be significantly suppressed and the CD206 level was up‐regulated, suggesting that RBAP promotes the shift from M1 to M2 polarization (Figure 2).

### 3.3 Effects of RBAP on microglia polarization markers

**FIGURE 3:**
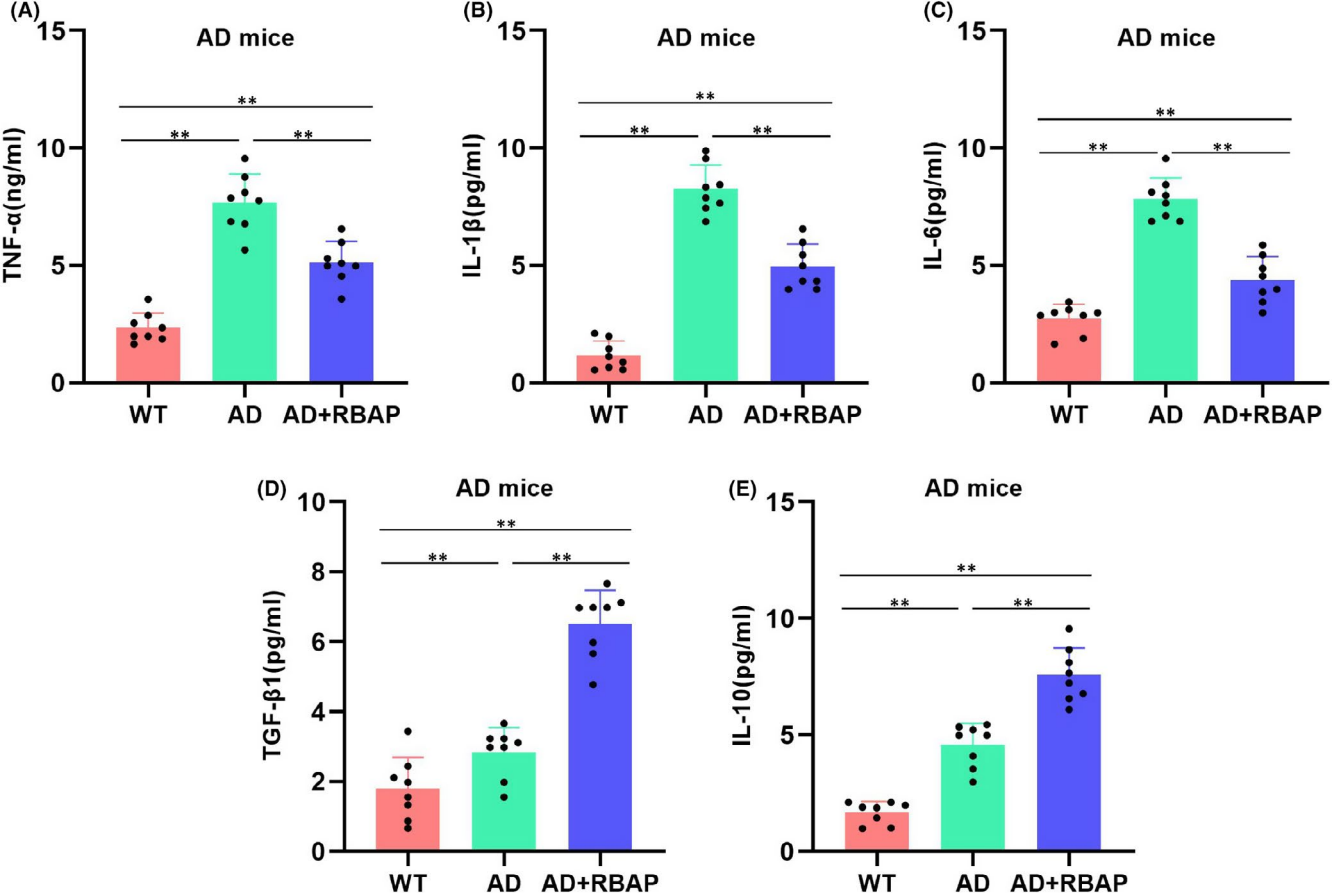
Effects of RBAP on the microglia polarization markers. (A‐C) Regarding the M1 marker expressions, the levels of TNF‐α, IL‐1β and IL‐6 were low in WT mice, while were up‐regulated significantly in AD mice. RBAP could inhibit the inflammatory cytokine expressions and reduce the TNF‐α, IL‐1β and IL‐6 levels. **p < 0.05. (D‐E) Regarding the M2 marker expressions, the levels of TGF‐β1 and IL‐10 were low in WT mice, while such expressions were slightly up‐regulated in AD mice. RBAP could significantly elevate the TGF‐β1 and IL‐10 levels. **p < 0.05

Our assessment of the cerebral tissue levels of inflammatory cytokines demonstrated that the levels of TNF‐α, IL‐1β and IL‐6 were low in WT mice, while such expressions were significantly up‐regulated in AD mice. M1 microglia predominated in AD mice, which promoted the inflammatory cytokine expressions significantly. RBAP could inhibit the inflammatory cytokine expressions and reduce the TNF‐α, IL‐1β and IL‐6 levels (Figure 3A‐C). During the detection of M2 marker cytokines, we found low levels of TGF‐β1 and IL‐10 in WT mice, while such expressions were slightly up‐regulated in AD mice. RBAP could significantly elevate the TGF‐β1 and IL‐10 levels (Figure 3D‐E).

## 4 DISCUSSION

Experimental research has shown that regulating the phenotypic changes of microglia in AD, inhibiting their activation towards M1 phenotype and inducing their shift from M1 to M2 polarization are all conducive to alleviating the immunoinflammatory responses, which thus reduce neuronal damage.^11^ Additionally, the TLR4‐mediated NF‐κB is also one major signal for activating M1 microglia.^12^ After activation by LPS and other factors, TLR4 promotes the substantial transcription and expression of inflammatory cytokines through P50 and P65 phosphorylation. Therefore, inhibiting TLR4 is one of the strategies for suppressing M1 microglia polarization.^13^

Polypeptides, compared to the small‐molecular chemical drugs, are highly specific and have less side effects, which are currently the focus of novel clinical drug development. Plants contain diverse bioactive polypeptides, of which RBAP is extracted from rice. Existing research has demonstrated that RBAP can target TLR4^8^ to inhibit the hydrogen peroxide‐induced endothelial cell injury, which is though seldom used currently. In this study, we focus on exploring the effect of RBAP on the microglia polarization. A combined intervention with LPS and IFN‐γ can induce the M1 polarization of BV2 cells while prominently up‐regulating the inflammatory cytokine expressions. Contrastively, RBAP intervention alone has insignificant effect on the BV2 cell polarization. Nevertheless, RBAP can significantly reduce the proportion of M1 cells and inhibit the inflammatory cytokine expressions in M1 cells. Besides, LPS can also induce the TLR4 expression. According to the IFC results, the CD86 and TLR4 levels in the L/I group are significantly up‐regulated. RBAP can suppress the TLR4 and CD86 levels, as well as the expression of ROS, a M1 marker.^14^ After activation by LPS, the ROS can also be used for regulating downstream NF‐κB. In our murine model, we found that RBAP can ameliorate the neurological functions and cognitive abilities of mice. In the Morris water maze test, RBAP ameliorated both the cognitive and motor abilities of mice, promoted the shift of cortical microglia from M1 to M2 polarization and inhibited the CD86 expression while up‐regulating the level of CD206, a marker of M2 cells. Meanwhile, RBAP also inhibits the microglia activation and reduces the inflammatory cytokine expressions. Noteworthy is that the expressions of TGF‐β1 and IL‐10, which are the M2 cell markers, are significantly up‐regulated, suggesting the ability of RBAP to regulate the polarization shift in animals. During pathological examination, we also found that RBAP can alleviate the neurocyte damage in AD mice.

## 5 CONCLUSION

In this study, RBAP, a plant‐derived TLR4‐targeting bioactive polypeptide, is found to inhibit the M1 polarization of microglia and promote their shift from M1 to M2 polarization, which thereby alleviates the neuroinflammation in AD. RBAP is expected to be a novel polypeptide drug for AD treatment.

## CONFLICT OF INTEREST

No Competing interests.

## AUTHOR CONTRIBUTIONS

**Liping Zhai:** Conceptualization (equal); Data curation (equal); Investigation (equal). **heping shen:** Formal analysis (equal); Methodology (equal); Project administration (equal). **yongjia sheng:** Methodology (equal); Resources (equal); Supervision (equal). **weiqun guo:** Data curation (equal); Formal analysis (equal); Validation (equal); Writing‐original draft (equal). **qiaobing guan:** Methodology (equal); Resources (equal); Validation (equal); Visualization (equal). **yu zhu:** Visualization (equal); Writing‐original draft (equal); Writing‐review & editing (equal).

## ETHICS APPROVAL AND CONSENT TO PARTICIPATE

The study was approved by Ethics Committee.

## CONSENT FOR PUBLICATION

All authors’ approval published the article.

## Data Availability

The data that support the findings of this study are available from the corresponding author upon reasonable request.
